# A Mixture of Baicalein, Wogonin, and Oroxylin-A Inhibits EMT in the A549 Cell Line *via* the PI3K/AKT-TWIST1-Glycolysis Pathway

**DOI:** 10.3389/fphar.2021.821485

**Published:** 2022-02-09

**Authors:** Hui-Juan Cao, Wei Zhou, Xiao-Le Xian, Shu-Jun Sun, Pei-Jie Ding, Chun-Yu Tian, Fu-Ling Tian, Chun-Hua Jiang, Ting-Ting Fu, Shu Zhao, Jian-Ye Dai

**Affiliations:** ^1^ Traditional Chinese Medicine College, North China University of Science and Technology, Tangshan, China; ^2^ School of Pharmacy, Lanzhou University, Lanzhou, China; ^3^ School of Biology and Food Engineering, Fuyang Normal University, Fuyang, China; ^4^ State Key Laboratory of Natural Medicines, China Pharmaceutical University, Nanjing, China; ^5^ Collaborative Innovation Center for Northwestern Chinese Medicine, Lanzhou University, Lanzhou, China

**Keywords:** epithelial–mesenchymal transition, reconstructed TFAE, stable isotope dimethyl-labeled proteomics, Twist1, PI3K/Akt signaling pathway

## Abstract

Non-small cell lung cancer (NSCLC) is a worldwide disease with a high morbidity and mortality rate, which is most derived from its metastasis. Some studies show that the epithelial–mesenchymal transition (EMT) process promotes lung cancer cell migration and invasion, leading to NSCLC metastasis. Total flavonoid aglycones extract (TFAE) isolated from *Scutellaria baicalensis* was reported to inhibit tumor growth and induce apoptosis. In this study, we found that baicalein, wogonin, and oroxylin-A were the active compounds of TFAE. After reconstructing with these three compounds [baicalein (65.8%), wogonin (21.2%), and oroxylin-A (13.0%)], the reconstructed TFAE (reTFAE) inhibited the EMT process of A549 cells. Then, bioinformatic technology was employed to elucidate the potential pharmacodynamic mechanism network of reTFAE. We identified the relationship between reTFAE and PI3K/Akt signaling pathways, with TWIST1 as the key protein. LY294002, the inhibitor of the PI3K/Akt signaling pathway, and knock-down TWIST1 could significantly enhance the efficacy of reTFAE, with increasing expression of epithelial markers and decreasing expression of mesenchymal markers in A549 cells at the same time. Furthermore, stable isotope dimethyl-labeled proteomics technology was conducted to complement the follow-up mechanism that the EMT-inhibition process may be realized through the glycolysis pathway. In conclusion, we claim that TWIST1-targeted flavonoids could provide a new strategy to inhibit EMT progress for the treatment of NSCLC.

## Introduction

Lung cancer is a worldwide disease with a high morbidity and mortality rate. According to the World Health Organization (2020), there were 2.207 million new lung cancer patients worldwide in 2020, only less than breast cancer, and lung cancer ranked first in the global causes of cancer-related death (World Health Statistics, 2020; Thai et al., 2021). Among the histological subtypes of lung cancer collectively, non-small cell lung cancer (NSCLC) accounts for 80%–85%, and most lung cancer patients are diagnosed at advanced stages, so traditional chemotherapy and radiotherapy have limited efficacy (Siegel et al., 2017). NSCLC microenvironment has limited nutrition, so cancer cells often obtain the energy and substances required for proliferation and cell growth through the “Warburg Effect” glucose metabolism changes (Gatenby and Gillies, 2004; Lopez-Lazaro, 2008), that is, cancer cells tend to produce energy through glycolysis rather than oxidative phosphorylation (Heiden et al., 2009; Muz et al., 2015; Eales et al., 2016). However, the metabolic change leads to further tumor cell growth, aggravation of epithelial–mesenchymal transition (EMT) process, and resistance to treatment. During the treatment of NSCLC, 40% of patients have metastases (Dwyer-Nield et al., 2017), and the main biological process is EMT (Tulchinsky et al., 2018). In the EMT process, cells gradually become spindle-shaped, losing the unique epithelial characteristics, intercellular adhesion, and cytoskeletal structure of epithelial tissue, and possessing polarity, individual migration ability, and invasion ability (Nieto et al., 2016). Cellular EMT process is related to TGFR, Wnt/*β*-Catenin, PI3K/Akt, Ras, MAPK, NF-κB pathways, and the transcription factors involved are TWIST (TWIST1 and TWIST2), Slug, ZEB (ZEB1 and ZEB2), and Snail (Shaw and Solomon, 2011; Otsuki et al., 2018). EMT transcription factors promote the development of drug resistance (Staalduinen et al., 2018; Liu et al., 2020). In the process of EMT, the activated PI3K/Akt pathway promotes glucose uptake and glycolysis (Semenza, 2010; Tennant et al., 2010), and phosphorylated Akt can phosphorylate TWIST1, then up-regulates the TGF*β*-Smad pathway, which further promotes the EMT process (Xue et al., 2012). TWIST1 was known to bind to the E-box sequence of E-cadherin and inhibited the expression of E-cadherin to induce EMT in a variety of tumors (Ren et al., 2016; Zhu et al., 2016; Ding et al., 2019). Inhibition of TWIST1 was known to induce growth inhibition and apoptosis of EGFR-mutant NSCLC cells (Yochum et al., 2019).

Some herbals and their extracts have been reported to have therapeutic effects on cancer, including *Scutellaria baicalensis* (Sato et al., 2013; Bie et al., 2017; Zhang et al., 2018; Hsiao et al., 2019), whose main components are flavonoids. We previously reported that Total Flavonoid Aglycones Extract (TFAE) isolated from *S. baicalensis* inhibited tumor growth and induced apoptosis, in which the main components, baicalein, wogonin, and oroxylin-A accounted for 41.4%, 13.3%, and 8.2%, respectively (Wang et al., 2016). Several studies have investigated the treatment of lung cancer with *S. baicalensis* components, including wogonin, baicalin, and oroxylin-A (Wei et al., 2016; Yu et al., 2017; Wang et al., 2021). This inspired us to reconstruct a compound combination with baicalein, wogonin, and oroxylin-A.

**FIGURE 1:**
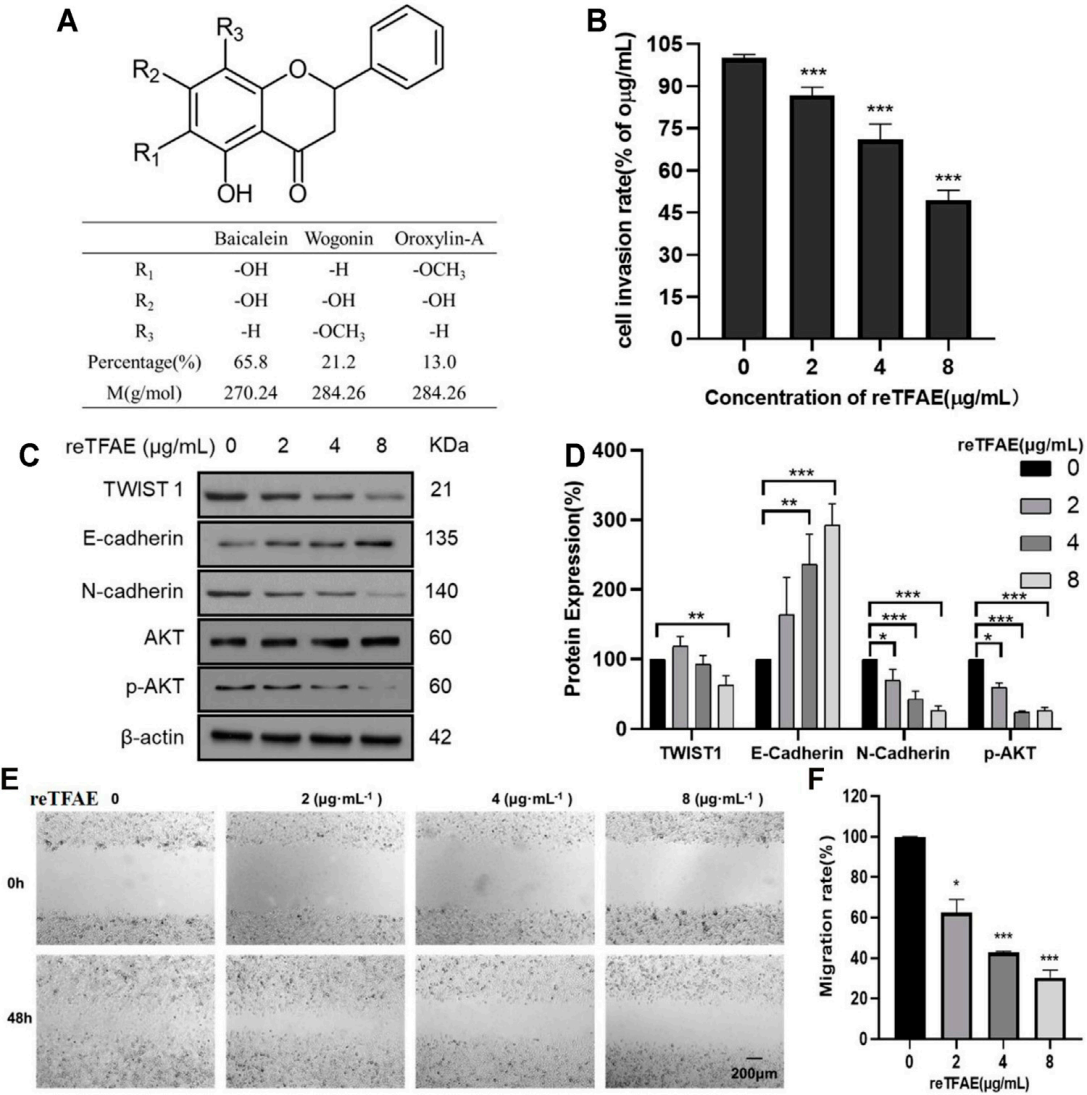
reTFAE inhibited EMT processes of A549 cells. **(A)** The structure and proportion of the components of reTFAE. **(B)** The invasion-inhibiting ability of reTFAE on A549 cells (*n* = 3). **(C,D)** Effects of different concentrations of reTFAE on the expression of EMT-related proteins (*n* = 3). **(E,F)** reTFAE inhibited A549 cells’ migration (*n* = 3). Statistical differences were determined by a two-sided Student’s *t*-test; Compared with 0 μg/μl TFAE. ***p < 0.001; **p < 0.01; *p < 0.05.

In this study, we reconstructed TFAE with baicalein (65.8%), wogonin (21.2%), and oroxylin-A (13.0%) and named it as reTFAE (Figure 1A), and found that the reTFAE could significantly inhibit EMT of A549 cells. We constructed the reTFAE-PI3K/AKT-TWIST1 correlation in inhibiting EMT progress, and revealed that this phenomenon may be related to glycolysis pathway through stable isotope dimethyl-labeled proteomics technology. Moreover, we proved that TWIST1 may be a potential target to inhibit the EMT process of lung cancer, and hope to explore a new combined medication strategy through the compatibility study of active compounds in herbal extracts.

## Materials and Methods

### Cell Culture

The A549 cells were obtained from the National Collection of Authenticated Cell Culture and were cultured at 37°C under 5% CO_2_ in Dulbecco’s modified Eagle’s medium (DMEM; Sigma, D6429) supplemented with 10% (vol./vol.) fetal bovine serum (FBS; Gibco, 10270-106) and 1% (vol./vol.) penicillin-streptomycin (PS; Gibco, 15140-122). Culture medium was refreshed and the cells were passaged when the confluency was 80%.

### Preparation of Cisplatin, LY294002, and reTFAE

As shown in Table1, 4 mg/ml cisplatin (Solarbio, D8810) stock solution, 20 mM LY294002 (AbMole, M1925) stock solution, and 128 mg/ml reTFAE solution were prepared by DMSO and were used for the later experiments. For reTFAE, the three main components and their mass percentages were baicalein (65.8%, Sichuan Weikeqi Biological, wkq-00289), wogonin (21.2%, Sichuan Weikeqi Biological, wkq-00245), and oroxylin-A (13.0%, Sichuan Weikeqi Biological, wkq-00180). We weighed the amount of each component according to their mass percentage, then mixed the three compounds, and dissolved them to the desired volume with DMSO.

**TABLE 1:** Compounds.

Compound	CAS number	Purity	Chemical name
Baicalein	491-67-8	≥98%	5,6,7-Trihydroxy-2-phenyl-4H-chromen-4-one
Wogonin	632-85-9	≥98%	5,7-Dihydroxy-8-methoxy-2-phenylchromen-4-one
Oroxylin-A	480-11-5	≥98%	5,7-Dihydroxy-6-methoxy-2-phenylchromen-4-one
LY294002	154447-36-6	99.84%	2-(4-Morpholinyl)-8-phenyl-4H-1-benzopyran-4-one
Cisplatin	15663-27-1	≥98.5%	trans-Dichlorodiamineplatinum (II)

### Wound Healing Assay and Cell Invasion Assay

For wound healing assay: 5.0 × 10^6^ A549 cells were plated in six-well plates. After 7 h, the cells formed a single layer with nearly 100% confluency. Confluent cell layers were scratched using 200-μl tips to generate wounds with approximately 800 µm width. Cells were washed three times with PBS to remove detached cells and debris. Cells were incubated with complete medium with or without 8 μg/ml reTFAE, 4 μg/ml Cisplatin, or 20 μM LY294002 for 24 h. At the 0th and 24th hour after scratching, three fields of view for each well were selected randomly to take pictures. ImageJ software was used to scan the scratch areas and to calculate the wound healing area. Wound healing rate = (wound area at 0th hour − wound area at 24th hour)/wound area at 0th hour × 100%.

For cell invasion assay (Bauer et al., 2005; Li et al., 2020; Nishida et al., 2020): the transwell containing polycarbonate membranes with 8 μm pore size was precoated with 50 µl of Matrigel (1.0 mg/ml) and incubated at 37°C overnight. A total of 1.0 × 10^6^ A549 cells and reTFAE (2, 4, and 8 μg/ml) in serum-free DMEM medium were plated in the upper chamber, and 150 µl of DMEM supplemented with 10% FBS in the lower chamber. After 24-h incubation, cells inside the upper chamber might pass through the matrix layer and transfer to the lower membrane surface. The upper chamber was taken out and washed two times by wash buffer. Cells in the upper surface of the membrane were completely removed with cotton swabs, and cells migrated to the lower membrane surface were fixed with 4% paraformaldehyde for 5 min. Then, the upper chamber was transferred to test board containing 100 µl Cell Dissociation Solution/Calcein-AM. After 1-h incubation, fluorescence value was detected at 485-nm excitation wavelength and 520-nm emission wavelength. Cell invasion rate = (number of invaded cells in experimental wells/number of invaded cells in control wells) ×100%.

### Western Blot

The A549 cells under different conditions were collected after washing three times by PBS and centrifugation at 8,600 rcf for 3 min. The cells were lysed in RIPA Lysis Buffer (CWBIO, CW2333S) containing EDTA-free cOmplete (Roche, 4693132001) with sonication on ice. The cell lysates were collected by centrifugation (2.15 × 10^4^ rcf, 20 min) at 4°C. The protein concentration was detected by Pierce™ BCA Protein Assay Kit (Thermo Fisher Scientific, 23225) and adjusted to 2 mg/ml. Twenty micrograms of the lysates was separated by 10% SDS-PAGE and then transferred onto a PVDF membrane in an ice bath at 300 mA for 1 h. The membrane was blotted with 5% nonfat milk in TBST at RT for 1 h and then incubated with the following antibodies at 4°C overnight: TWIST1 (1:1,000, Cell Signaling Technology, 46702S), E-cadherin (1:1,000, Cell Signaling Technology, 3,195), Vimentin (1:1,000, Cell Signaling Technology, 5,741), N-cadherin (1:1,000, Santa, Sc-59987), AKT (1:1,000, Cell Signaling Technology, 4,685), *p*-AKT (Ser473) (1:2,000, Cell Signaling Technology, 4,060), *β*-Actin (1:5,000, Proteintech, 20536-1-AP), and GAPDH (1:5,000, Proteintech, 10494-1-AP). Then, the membrane was incubated with secondary antibodies at RT for 1 h. The secondary antibodies are as follows: Goat anti-Rabbit IgG (1:5,000, Proteintech, SA00001-2) and Goat anti-mouse IgG (1:5,000, Proteintech, SA00001-1). Finally, the immunoreactive bands were visualized by the Clarity™ Western ECL Substrate (BIO-RAD, 1705061) using the Tanon4600 Automatic chemiluminescence image analysis system (Tanon, Shanghai, China). ImageJ software was used to analyze the thickness of protein bands. Relative expression of target protein = target protein band/*β*-Actin band. AKT phosphorylation level = p-AKT band/AKT band.

### Transfection With siRNA

A total of 6.0 × 10^5^ A549 cells were cultured in medium without PS and were plated in six-well plates. After 7 h, the cells with nearly 70% confluency were transfected with TWIST1 siRNA. The siRNA sequences were as follows: TWIST1 (sense: 5′-CAA​GAU​UCA​GAC​CCU​CAA​GTT-3′, antisense: 5′-CUU​GAG​GGU​CUG​AAU​CUU​GTT-3′); GAPDH (sense: 5′-AAT​GGG​CAG​CCG​TTA​GGA​AA-3′, antisense: 5′-TGA​AGG​GGT​CAT​TGA​TGG​CA-3′). Negative control siRNA, which were confirmed not to interact with any mRNA sequence else, was used to balance siRNA where necessary. siRNA sequences were designed and synthesized by GenePharma (Shanghai, China). The interference was performed based on the protocol of Lipofectamine^®^ 3,000 (Thermo Fisher Scientific, L3000150). In detail, for each well, 5 μl of 15-μm siRNA primers dissolved in 125 μl of opti-MEM (Gibco) was mixed with 7.5 μl of Lipofectamine^®^ 3,000 (Thermo Fisher Scientific, L3000150) dissolved in 125 μl of opti-MEM. After 20-min mixture, replace with 1 ml of new medium and add 250 μl of mixture dropwise to cells. After 8-h incubation, add 1 ml of medium and culture for another 48 h. The cells were collected separately and the knockdown efficiency was examined by qRT-PCR.

### Quantitative Real-Time PCR

Total RNA was extracted and purified with Eastep^®^ Super Total RNA Extraction Kit (Promega, LS1040) and 1 μg of total RNA was used as the cDNA synthesis template for reverse transcription with the RevertAid First Strand cDNA Synthesis Kit (Thermo Scientific, k1622). The reverse transcription was performed in 20 μl volume and the conditions were as follows: 42°C for 30 min; 85°C for 10 min. qRT-PCR was performed by MX3000P (Agilent) using UltraSYBR Mixture (Low ROX) (CWBIO, CW2601), and reaction was performed in 20 μl volume and the conditions were as follows: 95°C for 3 min; 95°C for 15 s; 55°C for 30 s, 72°C for 30 s; 40 cycles. The primers were as follows: TWIST1 (forward: 5′-TCG​GAC​AAG​CTG​AGA​GCA​AGA​TTC​A-3′, reverse: 5′-TCC​ATC​CTC​CAG​ACC​GAG​AAG​G-3′), GAPDH (forward: 5′-CAT​GAG​AAG​TAT​GAC​AAC​AGC​CT-3′, reverse: 5′-AGT​CCT​TCC​ACG​ATA​CCA​AAG​T-3′). CT values were used to evaluate the relative mRNA expression by 2^−ΔΔCt^ method and *β*-Actin served as an internal control. The primers were designed and synthesized by GenePharma (Shanghai, China).

### Protein In-Solution Digestion and Dimethyl Labeling

A549 cells were grown to 80% confluence in a 15-cm dish. In our previous experiments, 64 μg/ml reTFAE had a more stable and stronger EMT-inhibition effect than 8 μg/ml reTFAE. To obtain a more accurate and reliable proteomic result, A549 cells were treated with 64 μg/ml reTFAE for 24 h. The cells were collected after washing three times by PBS and centrifugation at 8,600 rcf for 3 min. The cells were lysed in 0.1% Triton X-100 (Sigma-Aldrich, T8787)-100 mM TEAB (Sigma-Aldrich, T7408) containing EDTA-free cOmplete with sonication on ice. The cell lysates were collected and the protein concentration was detected by Pierce™ BCA Protein Assay Kit. Ten-microliter cell lysates (3 mg/ml) were reacted with 30 µl of 8 M urea (Sigma, BCBZ1744) and 2 µl of 200 mM DTT (Sigma-Aldrich, 43815) at 65°C for 15 min in the dark. Then, 2 µl of 400 mM iodoacetamide (Sigma, I1149) was added to react in the dark for 30 min at 35°C. Two microliters of 200 mM DTT was added to react with the remaining iodoacetamide in the dark for 15 min at 65°C. One hundred microliters of 100 mM TEAB, 2 µl of 0.2 μg/μl trypsin (Promega, V528A), and 1.5 µl of 100 mM CaCl_2_ were added and the trypsin digestion was performed at 37°C overnight. For dimethyl labeling: for the control (“light”) group, the peptides were reacted with 6 µl of 4% CH_2_O (Sigma-Aldrich, F1635) and 6 µl of 0.6 M NaBH_3_CN (Sigma-Aldrich, 42077); for the reTFAE (“heavy”) group, the peptides were reacted with 6 µl of 4% ^13^CD_2_O (Sigma-Aldrich, 596,388) and 6 µl of 0.6 M NaBD_3_CN (Sigma-Aldrich,190020). The reaction was incubated at 22°C for 1 h. The reaction was quenched by 24 µl of 1% ammonia (Sigma-Aldrich, 221228) and 12 µl of formic acid (Sigma-Aldrich, F0507). The “light” and “heavy” samples were combined and the desalination was performed by Pierce™ C18 Tips (Thermo Scientific,87782). LC-MS/MS analysis.

Samples were analyzed by LC-MS/MS on Q Exactive series Orbitrap mass spectrometers (Thermo Fisher Scientific). In positive-ion mode, full-scan mass spectra were acquired over the m/z ratio from 350 to 1,800 using the Orbitrap mass analyzer with a mass resolution of 7,000. MS/MS fragmentation was performed in a data-dependent mode, of which the TOP 20 most intense ions were selected for MS/MS analysis with a resolution of 17,500 under HCD’s collision mode. The isolation window was 2.0 m/z units, the default charge was 2+, the normalized collision energy was 28%, the maximum IT was 50 ms, and dynamic exclusion was 20.0 s. Under +57.0215 Da’s cysteine modification, the LC-MS/MS data were analyzed by ProLuCID. The isotopic modifications were set as static modifications on the N-terminal of a peptide and lysins, and 28.0313 and 34.0631 Da were for light and heavy labeling, respectively. The CIMAGE software was applied for quantitation, proteins with an average ratio (light/heavy) above 1.5 were selected for further KEGG analysis.

### Network Pharmacology

In this study, the common mechanism of reTFAE in the treatment of lung cancer was studied based on network pharmacology. The active ingredients and related targets of reTFAE were integrated from TCMSP, BATMAN-TAM, STP, and Pubchem databases. The standard names of these targets were united by UniProt database. Targets of lung cancer were enriched through GeneCards, NCBI (Gene), Therapeutic Target Database, and DisGeNET (v7.0) databases. Then, the intersection targets of reTFAE and disease were obtained. The STRING network and the Cytoscape 3.7.2 were used to construct a protein–protein interaction (PPI) network, and the DAVID database was used to perform KEGG analysis. Then, Cytoscape 3.6.1 was used to build the “Ingredient-Target-Signal Pathway” network.

### Statistical Analysis

Student’s *t*-test was used to compare experimental data. We analyzed the data in GraphPad Prism (GraphPad Software), using the unpaired, two-tailed *t*-test module. Statistical significance was considered when a *p* value was below 0.05. *p < 0.05; **p < 0.01; ***p < 0.001. N.S., not significant.

## Results

### reTFAE Inhibited EMT Processes of A549 Cells

To explore reTFAE’s effects in the EMT process of lung cancer, A549 cells were treated with reTFAE (2, 4, and 8 μg/ml) for 24 h. Compared to 0 μg/ml reTFAE, the invasion rate of cells treated by reTFAE concentration-dependently decreased (Figure 1B). In addition, reTFAE dose-dependently inhibited the expression of TWIST1 and N-cadherin proteins, and reduced AKT phosphorylation level, while increasing the expression of E-cadherin (Figures 1C,D). Compared with the wound area at the 0th hour, the wound was dose-dependently healed by reTFAE at the 48^th^ hour (Figures 1E,F), which means that reTFAE inhibited the migration of A549 cells.

### reTFAE Inhibited the EMT Process *via* the PI3K/Akt Pathway

**FIGURE 2:**
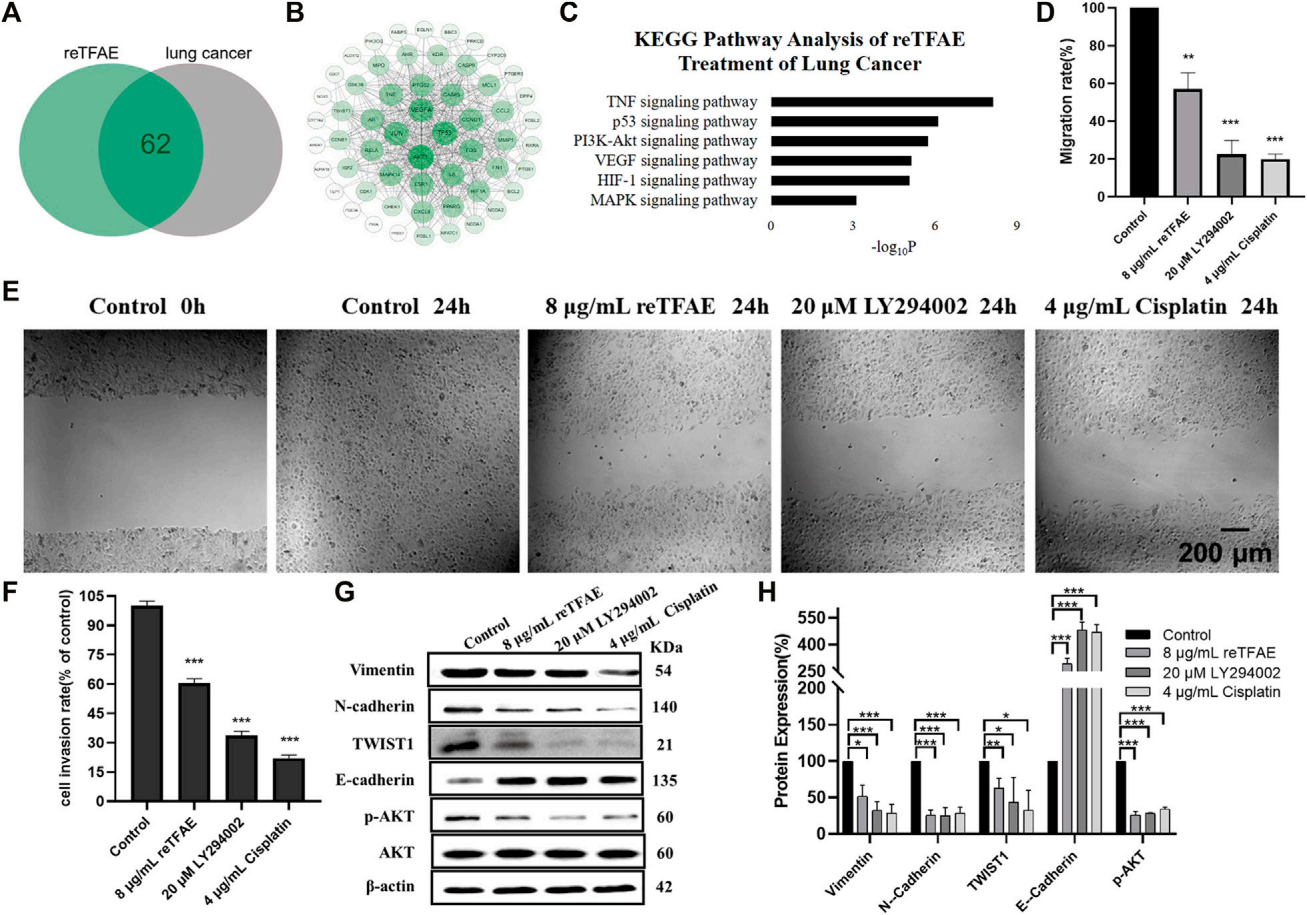
reTFAE inhibited the PI3K/Akt pathway to inhibit EMT processes of A549 cells. **(A)** Intersection protein targets of reTFAE and lung cancer. reTFAE targets and lung cancer targets shared 62 proteins. **(B)** Protein–protein interaction (PPI) network of 62 proteins. AKT1, TP53, VEGFA, and JUN showed more connections with other proteins. **(C)** KEGG pathway analysis of 62 proteins. *p* < 0.01 was the cutoff for displaying the pathways. **(D,E)** Both reTFAE and PI3K/AKT pathway inhibitors inhibited the migration of A549 cells (*n* = 3). **(F)** Both reTFAE and PI3K/AKT pathway inhibitors inhibited the invasion of A549 cells (*n* = 3). **(G,H)** The influence of PI3K/Akt-suppression and reTFAE on the expression of EMT-related proteins (*n* = 3). Statistical differences were determined by a two-sided Student’s *t*-test. Compared with control, N.S. not significant, ***p < 0.001; **p < 0.01; *p < 0.05.

The above results indicated that reTFAE exerted a concentration-dependent inhibitory effect on the EMT process of A549 cells. To explore the connection between reTFAE and lung cancer, network pharmacology analysis was performed. Three active ingredients and 65 related targets of reTFAE were integrated from TCMSP, BATMAN-TAM, STP, and Pubchem databases. The targets of lung cancer were enriched through GeneCards, NCBI (Gene), Therapeutic Target Database, and DisGeNET (v7.0) databases. Venn diagram showed the intersection of 62 reTFAE and lung cancer targets (Figure 2A). STRING network and Cytoscape 3.7.2 were used to construct the PPI network (Figure 2B); AKT1 was the central protein. DAVID database was used to perform KEGG analysis, reTFAE regulated pathways with *p* < 0.01 were as follows: TNF signaling pathway, p53 signaling pathway, PI3K/Akt signaling pathway, VEGF signaling pathway, HIF-1 signaling pathway, and MAPK signaling pathway (Figure 2C). After literature research, the PI3K/Akt signaling pathway attracted our attention, which could significantly activate tumor metastasis and directly regulate the expression of TWIST1, one of the key markers in EMT. To investigate whether reTFAE affected the EMT process of A549 cells by acting on the PI3K/Akt signaling pathway, we incubated A549 cells with the PI3K/Akt pathway inhibitor, LY294002. In this part, cisplatin was used as a positive control.

Compared with normal cells, inhibition of the PI3K/Akt pathway showed poor migration and invasion capabilities (Figures 2D–F), and increased expression of E-cadherin and decreased expression of Vimentin, N-cadherin and TWIST1, and reduced AKT phosphorylation level (Figures 2G,H), which were related with EMT progress. Therefore, we believed that reTFAE inhibited the EMT process of A549 cells by inhibiting the PI3K/Akt signaling pathway.

### reTFAE Inhibited TWIST1 in the EMT Process of A549 Cells

**FIGURE 3:**
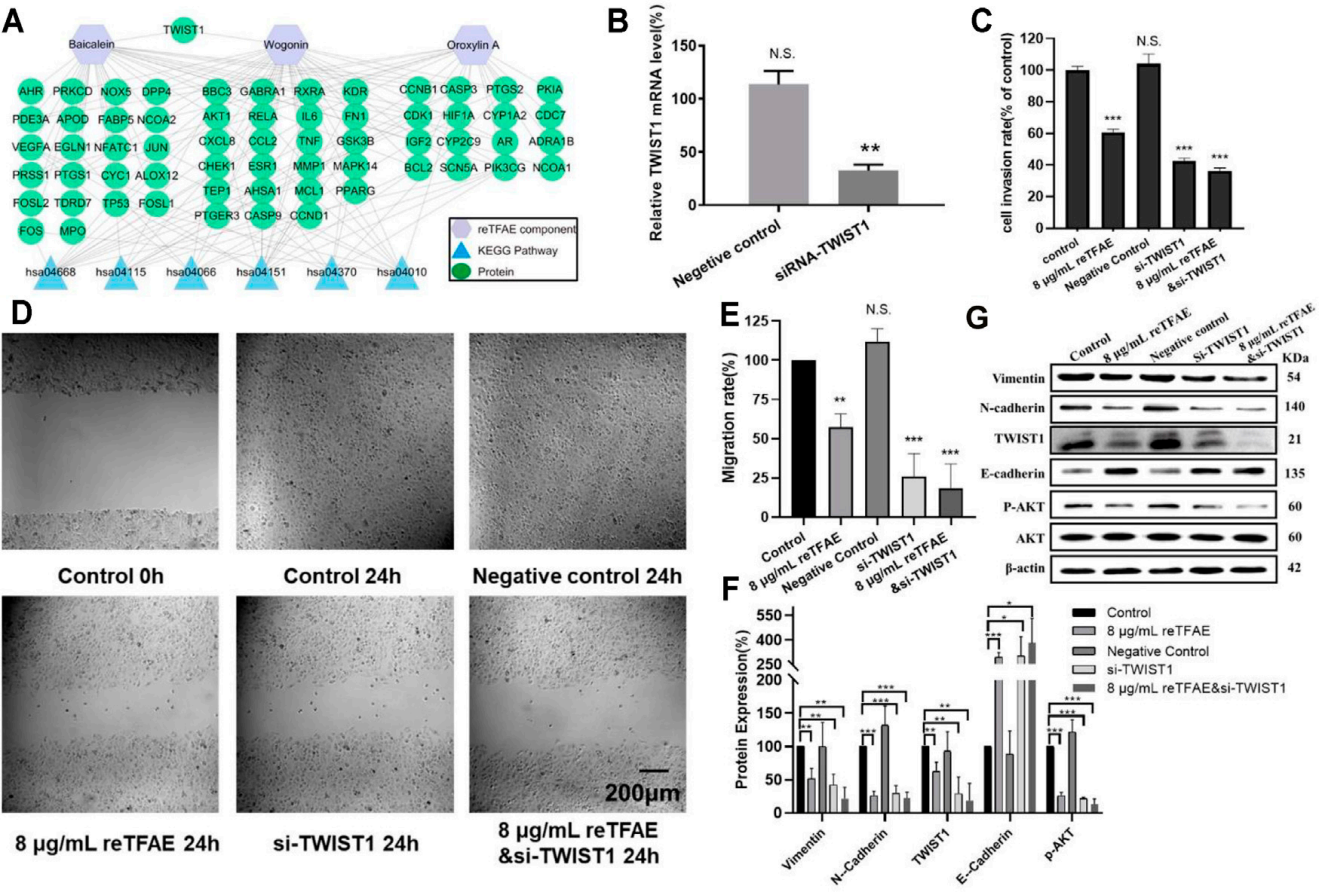
reTFAE inhibited the EMT process of A549 cells *via* inhibiting TWIST1. **(A)** Relationships of reTFAE components–KEGG pathways–Protein targets. **(B)** Relative TWIST1 mRNA level after siRNA interference (*n* = 3). **(C)**. The inhibitions of siTWIST1 and reTFAE on A549 cell invasion rate (*n* = 3). **(D,E)** The inhibitions of siTWIST1 and reTFAE on wound healing (*n* = 3). **(F,G)** The influence of siTWIST1 and reTFAE on the expression of EMT-related proteins (*n* = 3). Negative control siRNA was confirmed not to interact with any mRNA sequence else, and was used to balance siRNA where necessary. Statistical differences were determined by a two-sided Student’s *t*-test; Compared with control, N.S., not significant, ***p < 0.001; **p < 0.01; *p < 0.05.

To identify the key target of reTFAE, Cytoscape 3.6.1 was used to build the “Ingredient-Target- Pathway” network. Among proteins involved in the network, TWIST1 attracted our attention, which was reported to be directly regulated by PI3K-Akt and connected with both baicalein and wogonin (Xue et al., 2012; Yao et al., 2017; Yuan et al., 2020; Zeng et al., 2020) (Figure 3A). To investigate whether TWIST1 played an important role in reTFAE’s effect on the EMT process, we applied the small interfering RNA (siRNA) method to knock down the TWIST1 gene in A549 cells.

Compared with normal cells, TWIST1-knockdown A549 cells showed significant lower mRNA level (Figure 3B), and reTFAE-treated/siTWIST1/reTFAE and siTWIST1-treated cells showed poor migration and invasion capabilities, increased expression of epithelial marker (E-cadherin), and decreased expression of mesenchymal markers (Vimentin and N-cadherin), EMT-related transcription factor (TWIST1), and protein in the PI3K/Akt pathway (*p*-AKT) (Figures 3C–E). siTWIST1 showed similar effects to reTFAE, and the synergistic application of reTFAE and siTWIST1 showed a more obvious effect on EMT-related protein expression (Figures 3F,G). Therefore, we claimed that reTFAE inhibited the EMT process of A549 cells *via* inhibiting TWIST1.

### The Effect of reTFAE on Protein Expression of A549 Cells

**FIGURE 4:**
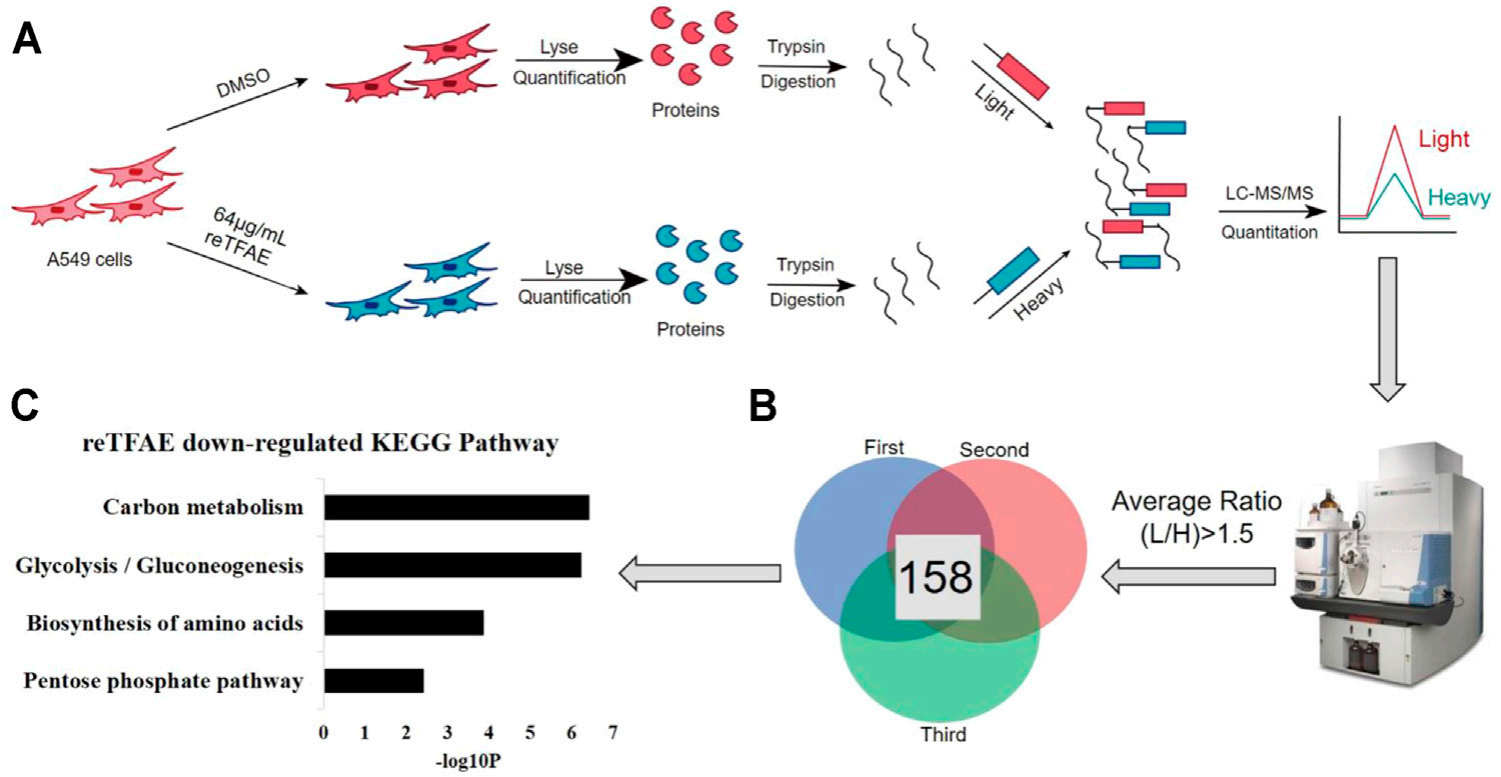
reTFAE interfered with the glucose metabolism of A549 cells. **(A)** Overall scheme of In-solution dimethyl labeling experiment, the control or reTFAE group were labeled with “light” or “heavy”, respectively. **(B)** Venn diagram showing the 158 proteins with an averaged ratio >1.5 appearing twice in triple experiments. **(C)** KEGG pathway analysis of the 158 proteins. *p* < 0.01 was the cutoff for displaying the pathways.

As above, we found that reTFAE inhibited the PI3K/Akt pathway and TWIST, yet how reTFAE led to the decline of EMT is not clear. To explain a further mechanism, we collected A549 cells treated with reTFAE for 24 h and performed a stable-isotope dimethyl labeling proteomics experiment. The proteins of A549 cells treated with DMSO or reTFAE were marked with “light” or “heavy”, respectively, and then mixed, desalted, and analyzed by LC-MS/MS (Figure 4A). Proteins with Average Ratio Light/Heavy (L/H) > 1.5 were filtered out, and 158 proteins downregulated by reTFAE appeared at least twice in repeated experiments (Figure 4B). KEGG pathway analysis was performed on the 158 proteins, and reTFAE downregulated pathways with *p* < 0.01 were as follows: Carbon metabolism, Glycolysis/Gluconeogenesis, Biosynthesis of amino acid, and Pentose phosphate pathway (Figure 4C). These four pathways contain 11, 9, 7, and four proteins, respectively (Table 1). In short, reTFAE interferes with the sugar metabolism of A549 cells.

## Discussion

As reported, TFAE of *S. baicalensis* has an inhibitory effect on NSCLC (Wang et al., 2016). In this study, we configured reTFAE and found that it inhibited the EMT process of A549 cells by affecting the PI3K/Akt pathway and TWIST1. As “knockdown TWIST1” and “reTFAE incubation” showed similar EMT-inhibition tendency, we believe that reTFAE inhibits the PI3K/Akt pathway and TWIST1, thereby inhibiting the EMT process of A549 cells. Actually, some evidence showed that the TWIST1-related axis may participate in the EMT process by activating the Wnt/*β*-catenin signaling pathway, thereby accelerating the process of lung cancer (Pan et al., 2020). TWIST1 promotes EMT and metastasis through serine phosphorylation of p38, c-Jun N-terminal kinase (JNK), and Erk1/2 (Jun et al., 2011). As our research provides direct evidence, TWIST1-targeted flavonoid may provide a new strategy to inhibit EMT progress.

Furthermore, stable-isotope dimethyl labeling proteomics was employed to detail the pharmacodynamic network of lung cancer cells treated with reTFAE. In this research, reTFAE downregulated the carbon metabolism, glycolysis/gluconeogenesis, biosynthesis of amino acid, pentose phosphate pathway, etc. Actually, tumor cells obtain energy mainly through the process of glycolysis, which promotes the EMT process of tumor cells (Liberti and Locasale, 2016); when nutrients were depleted, cancer cells tend to obtain metabolic materials through the pentose phosphate pathway (Georgakopoulos-Soares et al., 2020). Studies had shown that TWIST1 can promote the glycolysis process (Yang et al., 2015; Lovisa et al., 2020; Wang et al., 2020). In this study, ALDOA and GPI were enriched in both glycolysis and pentose phosphate pathways, while PKM and LDHA were enriched in glycolysis (Table2). The relationships between the above genes and the EMT process had been reported: PKM expresses pyruvate kinase and catalyzes the transfer of phosphorylation groups from phosphoenolpyruvate to ADP to generate ATP and pyruvate (Dombrauckas et al., 2005). PKM is expressed in fetal tissues and cancers, and participates in the EMT process of human colon cancer cells (Wong et al., 2015). PKM interacts with TGF*β*-induced factor homeobox 2 (TGIF2) to inhibit the transcription of E-Cadherin (Hamabe et al., 2014). Glucose-6-phosphate isomerase edited by GPI catalyzes the conversion of glucose-6-phosphate to fructose-6-phosphate. GPI can also be produced by cancer cells to promote the EMT process (Huang et al., 2019). GPI promotes the EMT process of breast cancer cells by inhibiting miR-200 and inducing ZEB1/2 (Teng et al., 2014). Silencing GPI promotes the transition of human lung fibroblasts from the mesenchymal to the epithelial state (Funasaka et al., 2007). ALDOA expresses fructose-bisphosphate aldolase A, which is involved in glycolysis and gluconeogenesis, and is also overexpressed in cancer (Chang et al., 2018). ALDOA is highly expressed in lung squamous cell carcinoma (LSCC) and depletion of ALDOA in lung squamous carcinoma cells reduces cell motility capabilities (Du et al., 2014). Overexpression of ALDOA in colon cancer cells leads to the EMT progress (Ye et al., 2018). In pancreatic cancer and bladder cancer cells, ALDOA-silencing increases E-Cadherin and decreases N-Cadherin expression (Ji et al., 2016; Li et al., 2019). Lactate dehydrogenase-A (LDHA) enzyme converts pyruvate into lactic acid. LDHA promotes the EMT process, but the specific mechanism is not clear (Hou et al., 2019; Tirpe et al., 2019). Our hypothesis is that reTFAE had a relationship with glycolysis or PI3K/Akt-related proteins to exert an EMT-inhibiting effect, yet it needs further verification.

**TABLE 2:** Pathways interfered by reTFAE, and proteins enriched in each pathway.

Pathways	Enriched targets
Carbon metabolism	GPI, PGAM1, PKM, HADHA, TALDO1, GAPDH, PGK1, MDH2, TPI1, ALDOA, G6PD
Glycolysis/Gluconeogenesis	GPI, PGAM1, PKM, LDHA, GAPDH, PGK1, TPI1, ALDH3A1, ALDOA
Biosynthesis of amino acid	PGAM1, PKM, TALDO1, GAPDH, PGK1, TPI1, ALDOA
Pentose phosphate pathway	GPI, TALDO1, ALDOA, G6PD

However, the details of the synergy between the three molecules need to be further studied. Therefore, next, we will explore the detailed pharmacodynamic mechanism of the three molecules.

**FIGURE 5:**
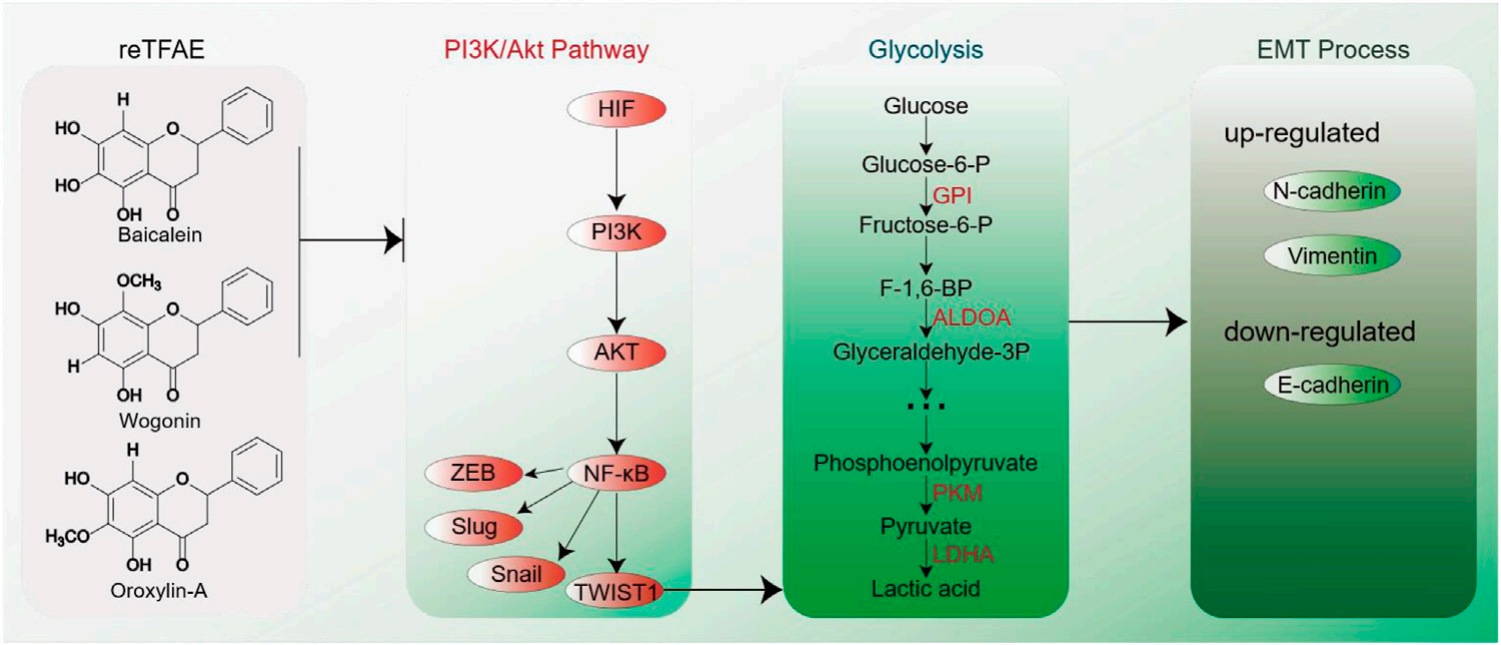
EMT process induced by reTFAE. Baicalein and wogonin inhibit the PI3K/Akt pathway, thereby inhibiting TWIST1, which inhibits the glycolysis pathway and inhibits the EMT process of A549 cells.

To conclude, in this work, we first determined that reTFAE composed of three compounds in a definite proportion had the activity of inhibiting the invasion and metastasis of lung cancer A549 cells. Then, combining with network pharmacology and molecular biology technology, the PI3K-AKT-TWIST1 axis was inhibited by reTFAE to inhibit EMT progress. Furthermore, chemoproteomics was used to elucidate the changes of downstream protein networks after TFEA inhibited the PI3K-AKT-TWIST1 axis, and it was found that the glycolysis pathway may play an important role. For clinical drug development, this study shows that multiple components can be used to treat lung cancer. Herbal medicine containing these three components can be used in the treatment of lung cancer. At the same time, the expression of EMT-related indicators in the body can be used as quality monitoring during the development of lung cancer treatment drugs. In summary, reTFAE can inhibit the PI3K/Akt pathway with the core of TWIST1, thereby inhibiting the glycolytic pathway to suppress EMT in A549 cells (Figure 5). TWIST1-targeted flavonoid provided a new strategy to inhibit EMT progress for the treatment of NSCLC.

## Data Availability

The datasets presented in this study can be found in online repositories. The names of the repository/repositories and accession number(s) can be found below: ProteomeXchange Consortium via the PRIDE partner repository with the dataset identifier PXD030097.
