# Supplementary material for: A Mixture of Baicalein, Wogonin, and Oroxylin-A Inhibits EMT in the A549 Cell Line via the PI3K/AKT-TWIST1-Glycolysis Pathway
Source: Front Pharmacol. 2022 Feb 9;12:821485. doi: 10.3389/fphar.2021.821485 (PMC8864075; doi:10.3389/fphar.2021.821485)

Supplementary Material

1.Western Blot pictures_ AKT:

AKT

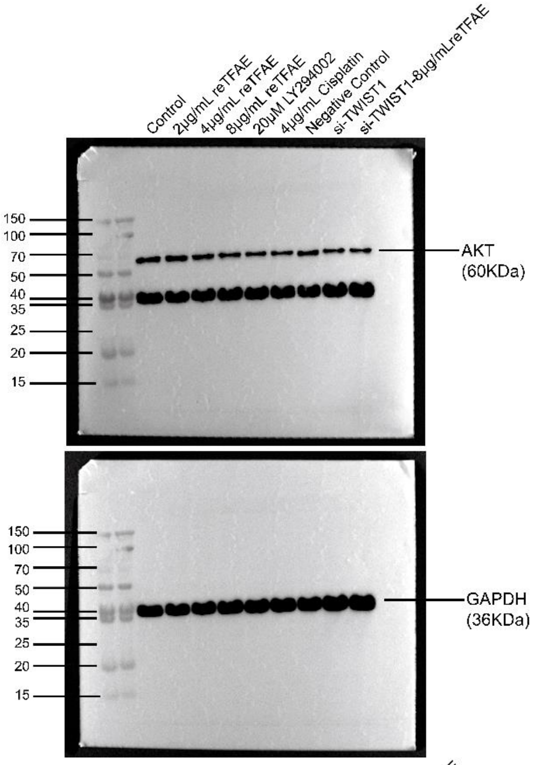

AKT

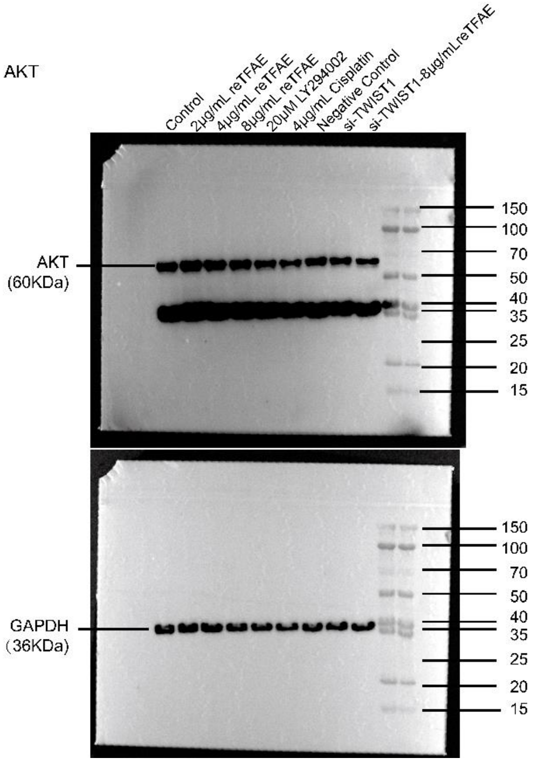

AKT

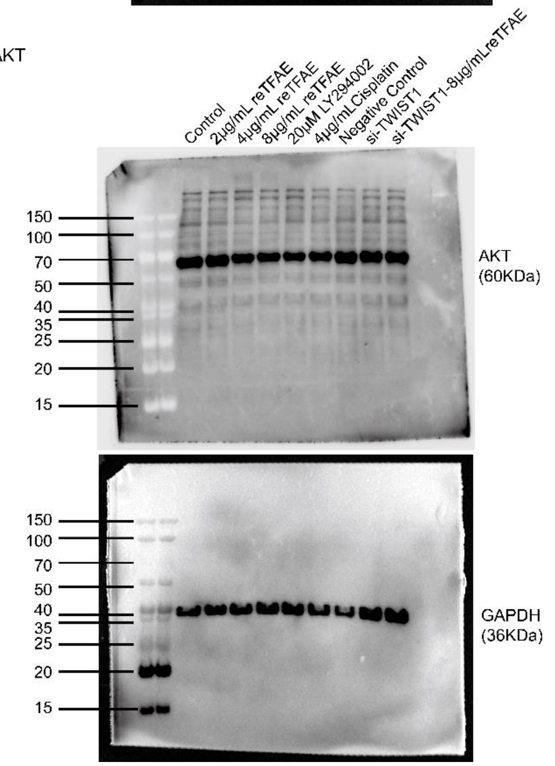

2.Western Blot pictures_ p-AKT:

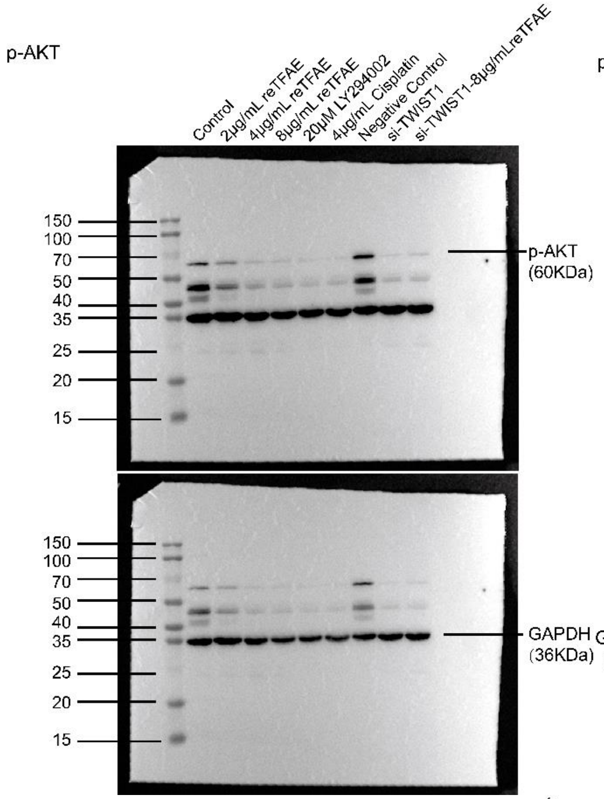

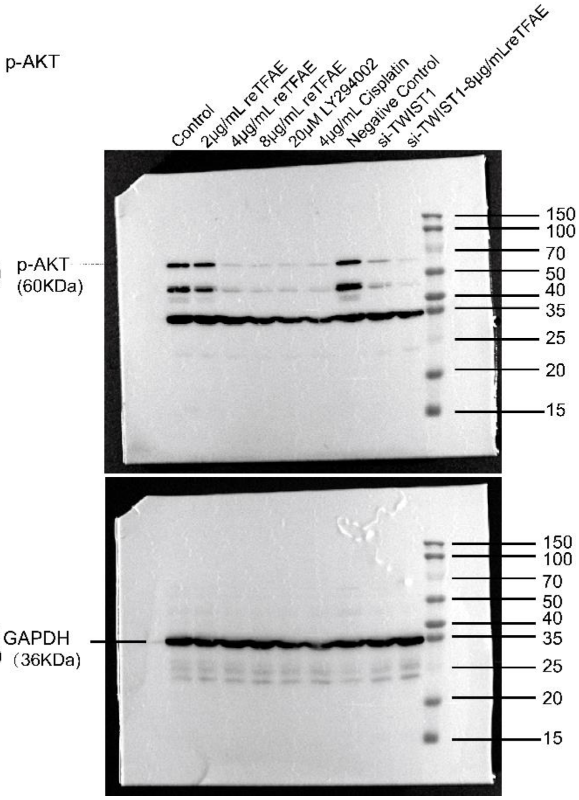

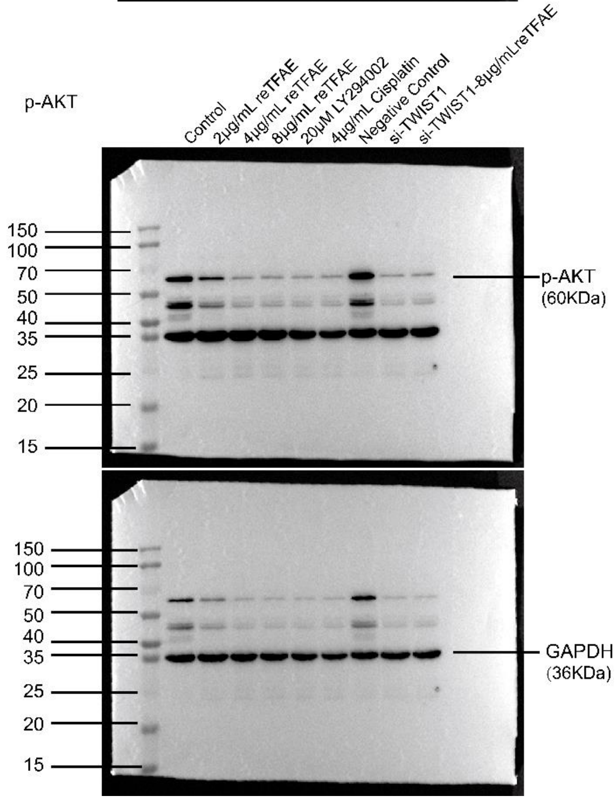

3.Western Blot pictures_ E-Cadherin:

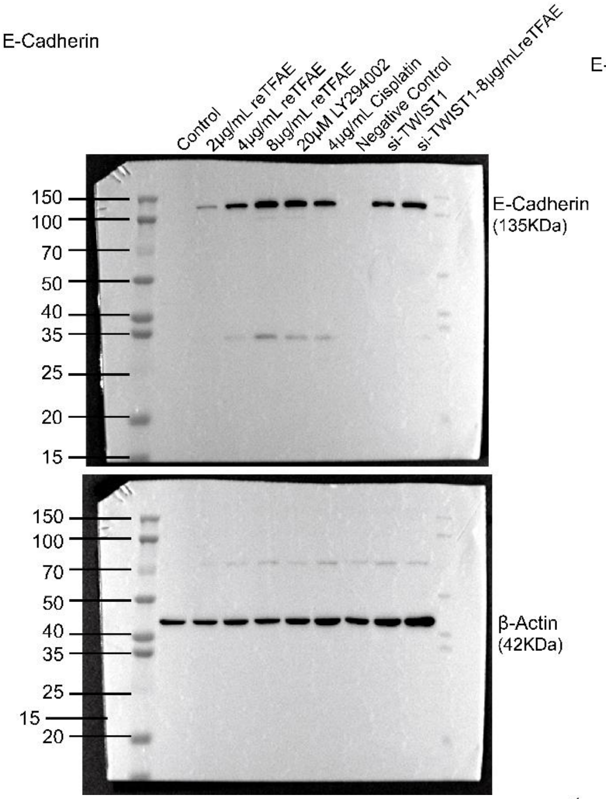

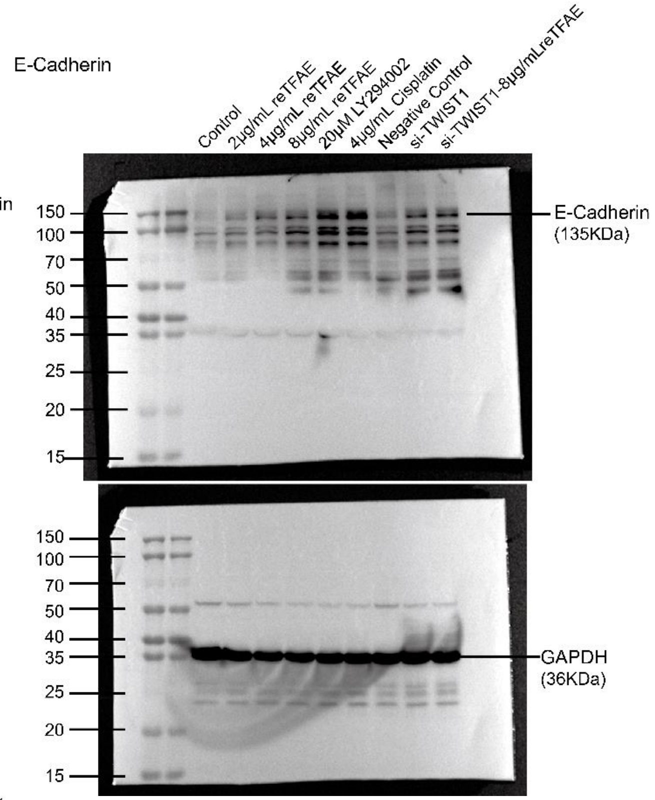

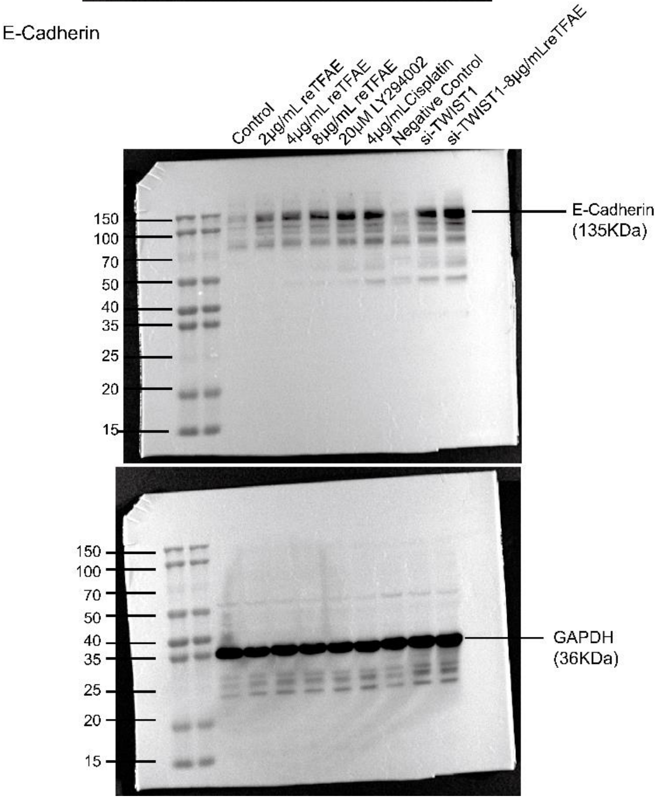

4.Western Blot pictures_ N-Cadherin:

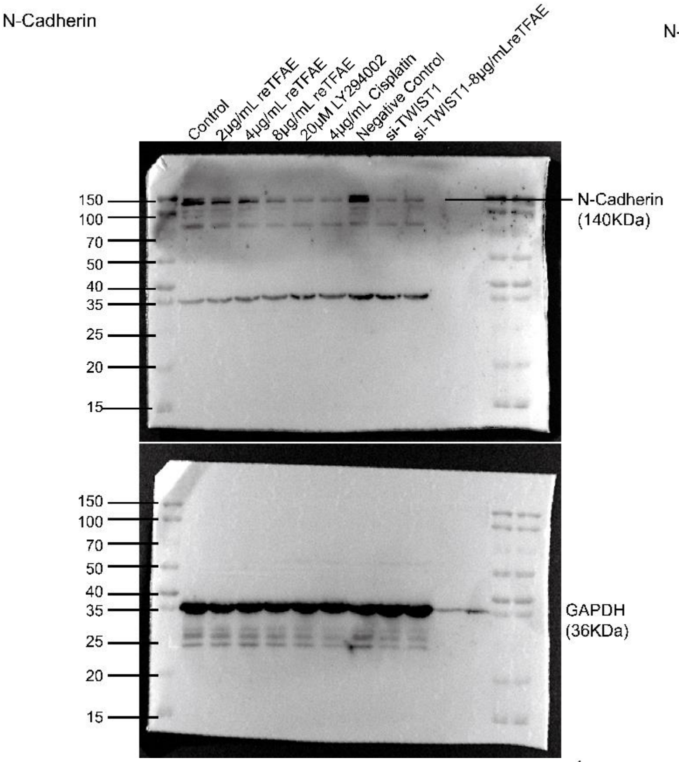

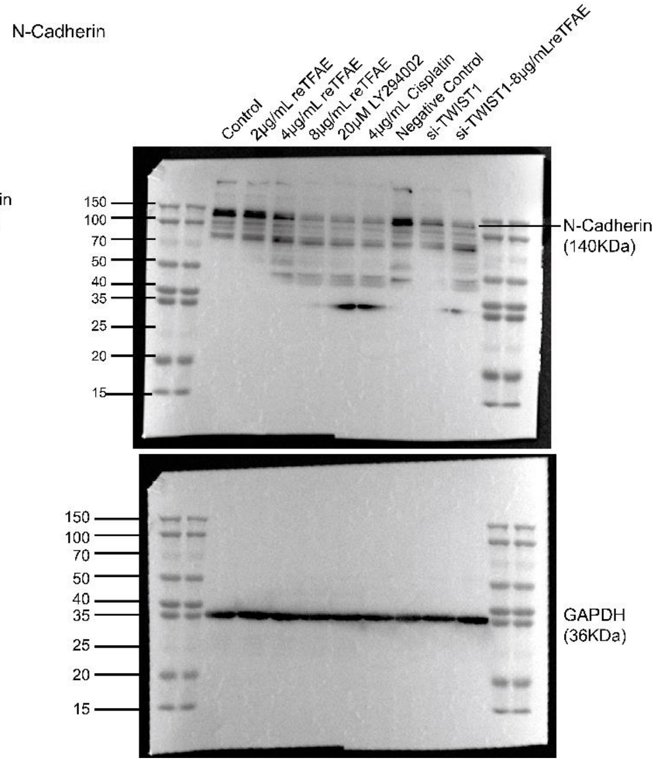

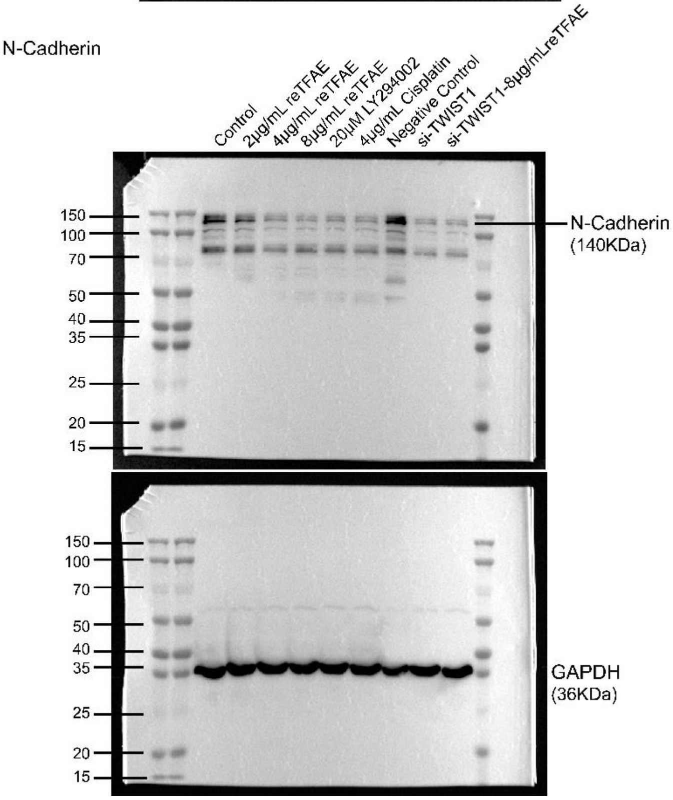

5.Western Blot pictures_ Vimentin:

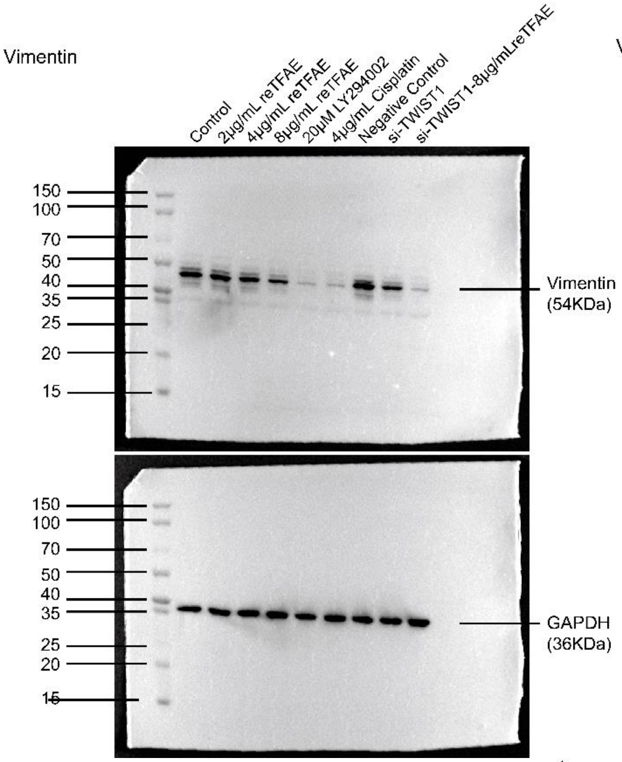

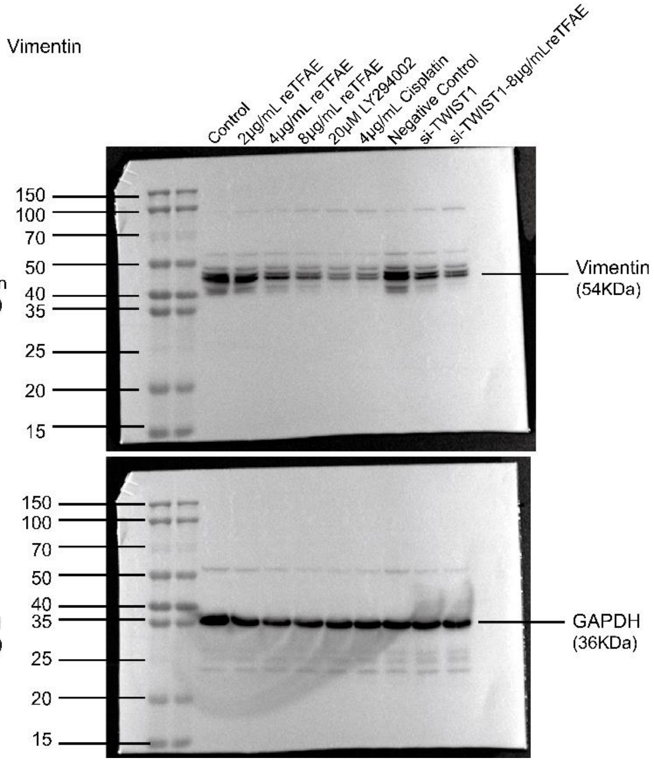

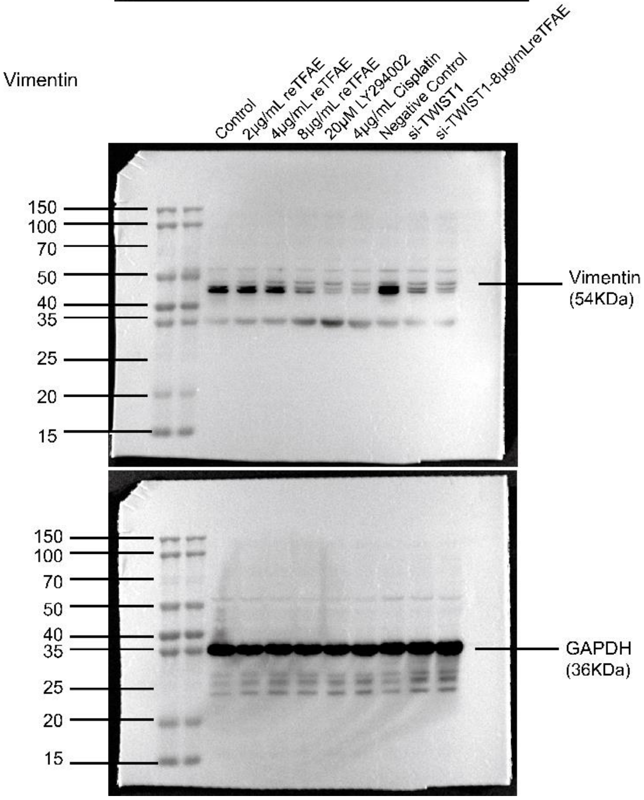

6. Western Blot pictures_ TWIST1:

TWIST1

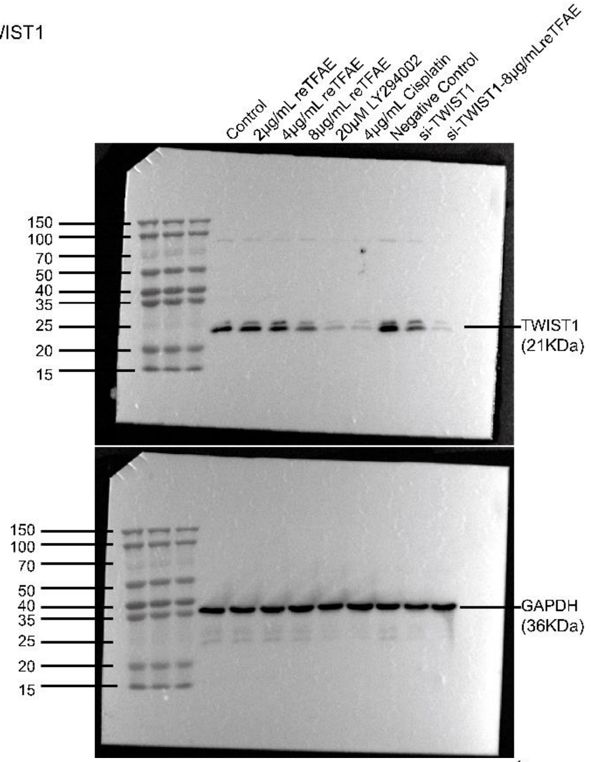

TWIST1

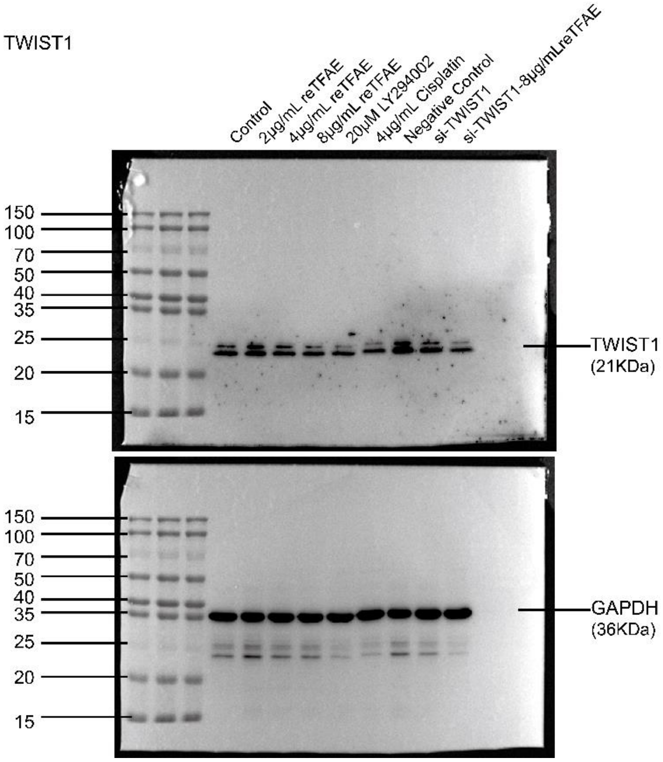

TWIST1

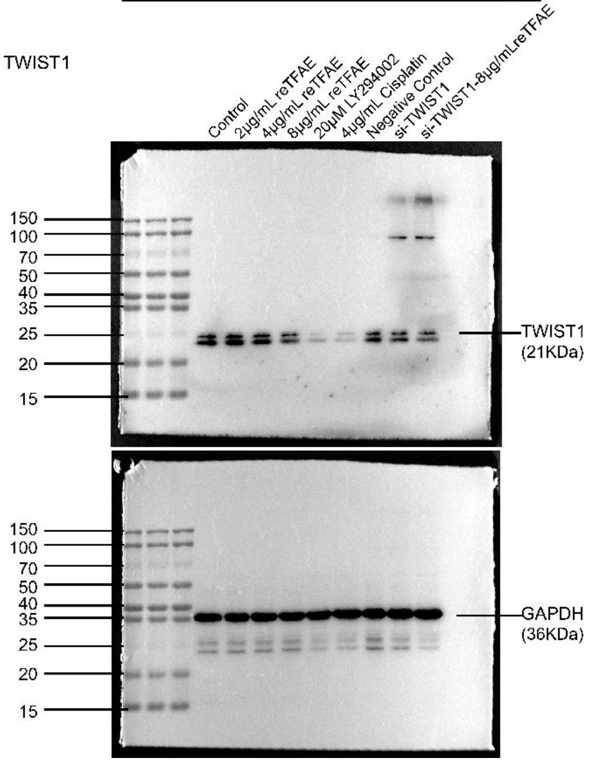

7. The effect of reTFAE on the viability of Beas-2B cells.

Beas-2B cells were obtained from the National Collection of Authenticated Cell Culture, and were cultured at 37°C under 5% CO_2 in Dulbecco's modified Eagle's medium (DMEM; Sigma, D6429) supplemented with 10% (vol./vol.) fetal bovine serum (FBS; Gibco, 10270-106), and 1% (vol./vol.) penicillin-streptomycin (PS; Gibco, 15140-122). Culture medium was refreshed and the cells were passaged when the confluency was 80%.

$5*10^3$ cells were plated in 96-well plates with 100 μL regular medium per well. After adherence, switch to medium without FBS while containing reTFAE or DMSO (as 0 μg/mL) for another 24 hours. After the treatment, remove the medium, and 60 μL of CCK-8 reagent (AbMole BioScience) prepared with serum-free medium was added to each well containing 1/11 original concentration of CCK-8. Incubate at 37°C within 4 hours, and measure the absorbance at 450 nm by a microplate reader. And the IC50 was 17.89 μg/mL (n=6).

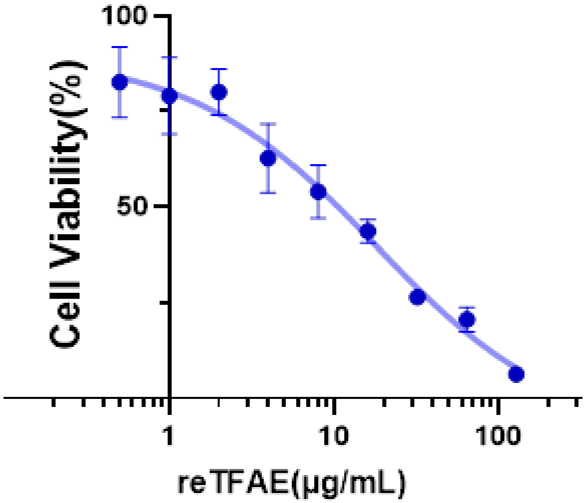

Supplement: Supplementary file 1 [file DataSheet1.pdf]
